# Revisiting the relation between oral contraceptive use and attentional engagement in everyday life: The role of formulation

**DOI:** 10.1371/journal.pone.0359652

**Published:** 2026-10-06

**Authors:** Alyssa C. Smith, Daniel Smilek

**Affiliations:** 1 Department of Psychology, University of Guelph, Guelph, Canada; 2 Department of Psychology, University of Waterloo, Waterloo, Canada; East West University, BANGLADESH

## Abstract

Oral contraceptives (OCs) are composed of artificial estrogen (ethinyl-estradiol) and/or progesterone (progestin), which can attach to receptors in the brain and potentially influence cognition. One prior study suggested that OC use was associated with increased mind-wandering – implying that OC use was associated with detriments to attention. In contrast, two subsequent well-powered conceptual replications of this initial finding revealed little evidence that OC use was related to increases in inattentiveness (including mind-wandering). However, these later studies collapsed across different OC formulations, which may obscure effects specific to particular formulations. In the present study, we investigated the possibility that OC formulation might differentially relate to attention-related traits. To do this, we collected trait-level reports of the tendency to experience attention related errors, attention lapses, spontaneous and deliberate mind-wandering, boredom, and flow in undergraduate females using OCs, and we collected the brand of OC used from which it was possible to derive the formulation. OC formulations differed in ethinyl-estradiol dose, progestin dose, dosing schedule, hormonal activity (or potency), and the type (or ‘generation’) of progestin used. We found no evidence that different OC formulations were related to differences in attention-related traits, which suggests that OC users can be treated as a homogenous group when examining trait-level attention. Comparisons of OC users (collapsing across formulation) to non-users again revealed no significant differences in trait-level attention.

## Introduction

More than 150 million women around the world use oral contraceptives [1]. Oral contraceptives are a type of hormonal contraceptive composed of ethinyl-estradiol and/or a synthetic form of progesterone known as progestin [2]. In naturally cycling individuals, estradiol and progesterone typically fluctuate over the course of the menstrual cycle. In contrast, OCs deliver a low consistent dose of synthetic versions of these hormones, suppressing the endogenous (or naturally occurring) estradiol and/or progesterone and replacing them with artificial surrogates [2]. This suppression of estradiol and/or progesterone (and replacement with artificial surrogates) is important because this process not only occurs in the reproductive system, but also systemically throughout the body. That is, estradiol and progesterone are also synthesized in areas of the brain responsible for higher order cognition (e.g., in the prefrontal cortex), which contain receptors for these hormones [3,4]. Since these molecules can function as neurosteroid hormones, they have the potential to modulate the neural excitability and neural circuits underlying many cognitive functions [5,6]. Accordingly, there has been a growing interest in gaining a better understanding of if and how the artificial hormones in OCs may be related to cognitive performance [7–9].

Reviews of the relations between OC use and cognition (see Warren et al [7]; Gurvich et al [8]; Beltz [9] typically categorize cognitive performance into several cognitive domains (e.g., memory, spatial perception, attention). One domain that had not been well examined is ‘attention’, and particularly processes related to sustained attention, such as mind wandering (i.e., engagement in task-unrelated thoughts). As of this writing, just two studies examined the relation between OC use and mind wandering [10,11].

In the first study addressing this issue, Raymond et al [10] examined self-reports of mind wandering among small samples of OC users (N = 28), OC non-users (N = 14), and men (N = 29). They claimed that OC users had an increased frequency of mind wandering compared to non-users. These results were in the expected direction given the increased risk of depression among OC users, and they were concerning, suggesting that OC use might be detrimental to attention. If this was in fact the case, women ought to know this risk when making the decision to begin using an OC. In the second study, we [11] aimed to replicate and extend the work of Raymond et al [10] with (1) a larger sample of participants, (2) validated measures of attentional engagement in everyday life (including a measure of mind wandering), and (3) a verification of our results across two large independent samples (OC users Sample 1: N = 471; Sample 2: N = 246; those not using any form of hormonal contraceptives including OCs, Sample 1: N = 1330; Sample 2: N = 929). Participants completed self-report measures of their trait-level tendency to experience spontaneous and deliberate mind wandering, attention lapses, and attention-related errors. In Sample 1, we found that compared to non-users, those using OCs reported a significantly lower propensity to experience spontaneous and deliberate mind wandering; there were also no differences between groups with regard to the tendency to experience attention lapses and attention-related errors. However, the effect sizes of the differences in mind wandering between groups were small in size (*d*s = 0.17, 0.20). In our second independent sample (Sample 2), we found no significant differences on any of our attention measures between groups. As such, given that the effect sizes in Sample 1 were small, and there were no significant effects observed in Sample 2, we concluded that OC use was not reliably associated with variations in trait-level attention [11].

While our results showed that the tendency to experience mind wandering, attention lapses, and attention-related errors was not reliably related to OC use, other attention-related traits could be. In a follow-up study we explored whether OC use was related to the tendency to experience boredom and flow [12]. Boredom is defined as an aversive state that arises when an individual is unable to successfully engage their attention with an internal (e.g., thoughts) or external activity, even though they may want to^24^. On the flip side, flow has been described as a state where individuals report being completely absorbed in a task where the full extent of their skills is used, while still being able to successfully complete that task. Most importantly, a side effect of this flow state is the lack of boredom or worry to the point where individuals can become oblivious to their surroundings [13]. While boredom and flow are often discussed as momentary states, there are also stable trait-level differences in how frequently individuals experience these states [13–16]. To examine the linke between OC use and these traits, we utilized a similar method to prior work examining attention-related traits. We found OC use was associated with decreased boredom proneness but no significant differences between groups in terms of internal or external flow. In hierarchical regressions predicting boredom, we entered depression, anxiety, and stress symptoms and the semester of data collection in the first step before adding OC use in the second step. This allowed us to examine the unique contribution of OC use by controlling for depression, anxiety, and stress symptoms and the semester of data collection. We found OC use was a unique and significant predictor of boredom proneness. This suggests that OC users and non-users differ in terms of boredom proneness, but that this difference is independent of mental health symptoms and the semester of data collection.

Taken together, our prior studies examining attentional engagement in everyday life and boredom and flow [11,12] revealed that OC use does not reliably relate to a variety of attention-related factors (i.e., attention-related errors, attention lapses, spontaneous and deliberate mind wandering, and internal and external flow), with the exception of the trait propensity to experience boredom [12]. One potentially important limitation of both studies was a lack of information about the formulations of the OCs being used by those in the OC group. OC brands are known to vary in the doses of the synthetic hormones they include, dosing schedule, and estrogenic, progestational, and androgenic activity. It is possible that different OC formulations may act differently on the brain and produce different behaviours as evidenced by different OC formulations being associated with varying risks of developing depression [17]. By not considering the various formulations of the OCs used, we may have obscured effects that are specific to particular formulations [18–20].

As an example of the possible moderating role of formulation is a study by Beltz et al [19]. They examined performance on a mental rotation task among women using OCs (with monophasic and triphasic dosing schedules), naturally cycling women, and men. When they collapsed across OC formulation, they found that women using OCs did not significantly differ from naturally cycling women, but when OC formulation was considered, women using monophasic OCs significantly differed from naturally cycling women such that monophasic OC users outperformed naturally women. In a separate study examining mental rotation, researchers again found that mental rotation performance did not significantly differ between OC users and non-users. However, they did find that the relation between self-perceived masculinity and performance on a mental rotation task among naturally cycling women and OC users was moderated by the estrogenic and progestational activity of OCs. This made us wonder if OC users and non-users may similarly differ in terms of attention-related traits once we accounted for the dosing schedule and/or the estrogenic, progestational, and androgenic activity of the OC used.

To address this limitation, in the present study we aimed to examine whether collapsing across OC formulation obscured differences in everyday attentional engagement. To do this, we collected data regarding OC brand as well as duration of OC use in order to determine whether there were effects of specific formulations on attentional engagement. Like our prior work, we focused on OC use (opposed to hormonal contraceptives more broadly) as prior work has suggested that the oral delivery of exogenous hormones could be associated with different metabolic and by extension, cognitive effects than other delivery methods [21]. As in our previous studies, we collected self-report trait-level measures of attention related errors, spontaneous and deliberate mind wandering, attention lapses, boredom proneness, and flow using reliable and validated questionnaires. While self-report measures do have limitations, there is also evidence that self-reports are better predictors of everyday behaviours than some task-based behavioural measures [22]. We aggregate data across five semesters (Fall 2023, Winter 2024, Spring 2024, Fall 2024, and Winter 2025) to achieve a larger sample.

Our main objectives were as follows: 1) replicate the overall null relations between OC use and everyday attentional engagement we reported previously [11,12]; and 2) then examine the role of OC formulation to determine whether collapsing across OC formulations obscured effects of specific formulations.

## Method

### Participants

This study utilized secondary data. All participants provided informed consent online (indicating their consent via the use of ratio buttons). Participants were recruited between September 5 2023 and April 1 2025. Participants in the secondary dataset were undergraduate students enrolled in at least one psychology course at the University of Waterloo who indicated their sex as female. In exchange for completing the Pre-screen and Mass Testing surveys (see below), participants received partial course credit. Since our goal was to determine whether our prior work [11] collapsing across OC formulation obscured differences in everyday attentional engagement, we aimed to collect data using the same recruitment techniques. Participants were then screened for the a priori determined exclusion criteria by semester based on the criteria outlined in the Data Cleaning section below. (We note that while a large proportion of participants were excluded, data was not collected for the sole purpose of the present study. Rather, the Prescreen and Mass Testing surveys are constructed for all researchers in the Psychology Department. As such, for the data to be utilized for the present study, inclusion criteria was applied following data collection.) Data were collected across 6 semesters: Fall 2023 sample (accessed November 15, 2023; N = 1546), Winter 2024 (accessed March 20, 2024; N = 1371), Spring 2024 (accessed July 26, 2024; N = 428), Fall 2024 (accessed November 27, 2024; N = 1554), and Winter 2025 sample (accessed April 2, 2025; N = 1459). Exclusion criteria and exclusion Ns are listed in Table 1. The total sample included in the data analysis collapsed across all semesters included a total of 2578 participants, with 355 OC users and 2223 were non-users. The total sample split by semester and OC status are included in the bottom of Table 1.

**Table 1:** Exclusion criteria and Ns of excluded participants by semester.

Exclusion criterion	Fall 2023	Winter 2024	Spring 2024	Fall 2024	Winter 2025
Previous semester inclusion	N/A	N = 345	N = 135	N = 442	N = 507
***Contraceptive use items***					
Other hormonal contraceptive use (not OC)	N = 17	N = 16	N = 4	N = 16	N = 13
IUD use	Copper N = 5, Hormonal N = 73	Copper N = 8, Hormonal N = 46	Copper N = 3, Hormonal N = 16	Copper N = 6, Hormonal N = 56	Copper N = 14, Hormonal N = 56
Non-response to hormonal contraceptive/ IUD use item	N = 39	N = 30	N = 16	N = 30	N = 29
Pregnant and/or breastfeeding	N = 160	N = 138	N = 40	N = 160	N = 0
Non-response to pregnant and/or breastfeeding item	N = 38	N = 17	N = 10	N = 28	N = 32
***Mental health items***					
Receiving treatment for depression	N = 148; OC N = 50, non-OC N = 98	N = 105; OC N = 24, non-OC N = 81	N = 45; OC N = 12, non-OC N = 33	N = 122; OC N = 27, non-OC N = 95	N = 121; OC N = 40, non-OC N = 81
Receiving treatment for anxiety	N = 75; OC N = 22; non-OC N = 53	N = 57; OC N = 17, non-OC N = 40	N = 18; OC N = 3, non-OC N = 15	N = 82; OC N = 25, non-OC N = 57	N = 83; OC N = 16, non-OC N = 67
Non-response to depression/ anxiety treatment item	N = 24	N = 10	N = 8	N = 21	N = 14
***Data quality checks***					
Too few clicks	N = 0	N = 0	N = 0	N = 0	N = 0
Speeded responses to survey items	N = 61	N = 76	N = 30	N = 59	N = 43
Responded to fewer than 50% of items on a survey	N = 4	N = 4	N = 0	N = 3	N = 4
***Age items***					
Age > 45 years old	N = 3	N = 5	N = 2	N = 4	N = 8
No response to year of birth item	N = 3	N = 2	N = 0	N = 1	N = 2
***Total Participants Excluded***					
	N = 650	N = 859	N = 315	N = 1030	N = 926
***Total Participants Included***					
OC group	N = 151	N = 52	N = 9	N = 77	N = 66
Non-OC group	N = 745	N = 460	N = 104	N = 447	N = 467

### Pre-screen and Mass Testing Surveys

Data was collected at the University of Waterloo as a part of the Pre-screen and Mass Testing surveys, which are questionnaires completed the beginning of each semester by undergraduates enrolled in psychology courses. The Pre-screen survey included items asking about sex, gender, age, current hormonal birth control use, length of current birth control use (only 35 participants provided a clear response in terms of the length of time they had used OCs. As such, we were underpowered to length of use as a mediator or moderator in the subsequent analyses. Exploratory analyses revealed no significant correlations between length of use and our attention measures: all ps > .062), current hormonal birth control brand name, past hormonal birth control use, length of past hormonal birth control use, time since past hormonal birth control use, and current pregnancy/breastfeeding. The hormonal birth control item was, “Are you currently using one of the following methods of birth control?” Participants responded by selecting from a list that included: oral contraception (i.e., birth control or “the pill”), birth control patch, vaginal ring, birth control injection, copper IUD, hormonal IUD, hormonal implant, none of the above, and prefer not to answer. We used the OC brand provided by participants to derive the composition (the type and dose of ethinyl estradiol and/or progestin), the dosing schedule (monophasic, triphasic, or progestin only), as well as the estrogenic, progestational, and androgenic activity of each OC [23]. Importantly, since data were collected as part of a larger test battery, all participants were blind as to the research question.

### Data Cleaning

We utilized a similar data cleaning procedure as in our prior work [11,12,24]. Exclusion criteria were determined a priori unless otherwise stated.

For all semesters, participants were excluded based on the following criteria: 1) inclusion in a previous semester of data (to ensure no participants were double counted; not applicable for Fall 2023 as the first semester of data collection), 2) use of a hormonal contraceptive other than OC, 3) use of an IUD, 4) failing to disclose whether or not they used a hormonal contraceptive, 5) current pregnancy or breastfeeding (due to a coding error in Winter 2025, the response options for the item asking participants whether they were pregnant or breastfeeding only included “no”. However, participants were not required to respond to all items. Since the inclusion criteria for this item is “no”, we reasoned that those who indicated no were indicating they were not pregnant or breastfeeding, while those who did not respond may have been pregnant or breastfeeding. As such, like the previous samples we only included those who responded “no”.), and 6) failing to disclose whether or not they were pregnant or breastfeeding. To avoid the possible confound of mental health, participants who reported that they were currently receiving treatment for depression or anxiety were excluded (the exogenous hormones in OCs could also interact with psychotropic medications; see Damoiseaux et al [25]). Participants who did not respond to the items related to the reception of treatment for depression and anxiety were also excluded. Data quality checks were conducted to eliminate poor quality data based on participants’ patterns of responses. Participants were removed prior to analyses if a) the number of clicks they made was fewer than the number of responses per page of a survey, b) their responses were faster than 1-second per scale item on more than 50% of the scales of interest (to respond in good faith, we reasoned participants needed at least 1-second per item to read and respond. If they were faster than this on >50% of the scales we removed them because this may indicate they were responding randomly), and c) participants responded to fewer than half of the items on a scale of interest (as responses on items for a given scale were averaged to calculate scale scores). We analyzed the data with and without exclusions based on age (to target participants of reproductive age; 18–45 years old). This did not change the results in a meaningful way. The below analyses exclude participants who were older than 45 or did not report their year of birth.

## Materials

### Spontaneous (MWS) and deliberate (MWD) mind wandering scales

The MWS is a self-report measure of spontaneous (or unintentional) mind wandering, while the MWD measures deliberate (or intentional) mind wandering [26]. These questionnaires are composed of 4 statements each. Example items include: “I allow my thoughts to wander on purpose” (MWD), and “I find my thoughts wandering spontaneously” (MWS). Participants are asked to rate each item on a seven-point scale that ranges from 1 (rarely) to 7 (a lot). Higher scores indicate a greater tendency to engage in mind wandering (deliberate or spontaneous) in everyday life.

### Mindful attention awareness scale-lapses only (MAASLO)

The MAASLO [27] was adapted from the MAAS [28] and includes 12 self-report items related to mindlessness or attention lapses in everyday life. Participants rate items such as “I rush through activities without being to really attentive them” on a scale from 1 (almost never) to 6 (almost always). Higher frequencies of attention lapses are indicated by higher scores on the scale.

### Attention-related cognitive errors scales (ARCES)

The ARCES assesses the propensity to experience performance errors as a result of attention lapses [27,29]. Participants respond to items such as “I have to go back to check whether I have done something or not (e.g., turning out lights, locking doors)” on a scale from 1 (never) to 5 (very often). Higher scores indicate a greater frequency of cognitive errors.

### Short Boredom Proneness Scale (SBPS)

SBPS assesses the tendency to experience boredom [16]. Participants respond to 8 statements such as “I find it hard to entertain myself” and “In most situations, it is hard for me to find something to do or see to keep me interested”. Each item is rated based on how typical the statements are of themselves on a 7-point scale that included anchors from 1 (strongly disagree) to 7 (strongly agree). Higher scores indicate a greater tendency to experience boredom.

### Deep, effortless concentration internal (DECI) and Deep, effortless concentration external (DECE)

The DECI measures the tendency to experience flow during internal tasks (such as thinking or remembering) and the DECE assesses the tendency to experience flow during external tasks (such as sports or hobbies) [15]. Each scale consists of eight items. Participants rate the frequency with which they experience internal and external flow. They respond to statements such as “When thinking/performing an external task, I get completely engaged without having to work at it”. Participants rate each item on a 7-point scale from 1 (never) to 7 (always). Higher scores indicate a greater frequency of flow experiences during internal or external tasks.

### Depression anxiety stress scale-21 (DASS-21)

The DASS-21 is a 21-item measures of depression, anxiety, and stress symptoms over the previous week [30]. Example items include “I felt that I had nothing to look forward to” (depression), “I was worried about situations in which I might panic and make a fool of myself” (anxiety), and “I found it hard to wind down” (stress). Participants rate each item a scale from 0 (did not apply to me at all) to 3 (applied to me very much or most of the time). The scale consists of seven items related to each of depression, anxiety, and stress and higher scores on the scale items indicate higher levels of these experiences.

### OC formulations

In order to conduct our analyses by OC formulation, we collected the brand participants were using via the Pre-screen survey. Past work (e.g., [20,31]) has used information reported by Dickey [23] to link OC brand names with composition, however, Dickey [23] lists the United States’ Food and Drug Administration (FDA) approved OCs, some of which have different names in Canada. To identify the formulation and dosing schedule associated with a brand name, we conducted an internet search (using Google) for each brand name with the word “monograph” to locate each OC’s product monograph (a drug monograph is a factual and scientific document that lists its name, classification, pharmacology, and indication, as well as dosage and administration [32].This is usually available through the pharmaceutical manufacturers website), which contained each OCs’ formulation and dosing schedule. In most cases, the ethinyl estradiol dose was noted in micrograms (mcg), while the progestin dose varied between milligrams (mg) and mcg. To ensure all doses were on the same scale, we converted progestin doses in mg to mcg (1 mg = 1000 mcg). Once we had the formulation and dosing schedule, we used the information reported by Dickey [23] in Table 5 to identify the formulation equivalent (by matching the dose, dosing schedule, and type of ethinyl estradiol and/or progestin). Since OCs have undergone four major formulaic iterations (“generations”) in the type of progestin used, we also sought to account for this variance in composition. The generations differ from one another in their molecular structure (the addition or removal of methyl or ethyl groups) which alter the biological activity of the progestin. Since earlier progestins (generations 1–3) were synthesized from testosterone, they have both progestational and androgenic activity (i.e., they can bind to receptors for both progesterone and testosterone), while more modern progestins (generation 4) are synthesized to be anti-androgenic (i.e., made to bind specifically to progesterone receptors while preventing binding to testosterone receptors [23] (pp 20 –21]). To identify these generations, we referred to Dickey [23] (pp20–21), which noted the progestin name and corresponding generation. While generations can be helpful to better understand the activity of OCs, the estrogenicity, progestational, and androgenicity of each formulation can be examined on a finer, more continuous scale by taking into account the dose and potency of hormones used in each formulation. These hormonal activities were determined from assays conducted in human and rodent tissue [23] (estrogenic activity was derived from mouse uterine assays, progestational activity from endometrial responses in humans, and androgenic activity from rat prostate assays [23]). These activity values can also be ranked as low, intermediate low, intermediate high, or high [23].

### Data analysis plan

Our analyses consist of two parts: First, we will compare OC users who reported their brand to OC users who did not report brand to determine if we can collapse these groups into one OC group. This will allow us to conduct planned comparisons of OC users to non-users in order to test the replicability of our prior findings [11,12]. Second, we will conduct analyses examining the link between OC composition and our trait-level attention measures to determine whether particular formulations are related to differences in attention. This will allow us to determine whether collapsing across OC formulation obscures effects of specific formulations.

## Results

All analyses were conducted using R [33]. To perform Null Hypothesis Significance Tests we used R (version 4.4.1) and utilized the psych, stats, Hmisc, ez, and basic R packages. Anonymized data and analysis scripts are available at: https://osf.io/myad4/overview

### Descriptive statistics

Descriptive statistics for each measure as a function of group (OC vs. Non-OC) are presented in Table 2. Since data was collected across multiple semesters, we also provide descriptive statistics as a function of group and semester of data collection in Supporting Information. All scales showed high reliabilities, with Cronbach alphas of.79 or greater (Table 3; left panel). Pearson correlations between the measures within each group are provided in Table 3 (right panel). We provide correlation coefficients split by OC status as these will inform the directions of the later regression analyses.

**Table 2:** Descriptive statistics of measures by OC status.

Group	Measure	N	Mean	SD	Skew	Kurtosis
Non-OC group	ARCES	2223	3.12	0.70	0.22	0.23
	MAASLO	2223	3.43	0.80	−0.11	0.34
	MWS	2223	4.64	1.31	−0.34	−0.08
	MWD	2223	4.73	1.42	−0.37	−0.30
	SBPS	2223	3.60	1.16	0.17	−0.48
	DECE	2223	4.08	1.24	0.06	−0.32
	DECI	2223	4.07	1.19	0.10	−0.21
	DASS-Dep	2223	0.95	0.76	0.66	−0.45
	DASS-Anx	2223	0.91	0.69	0.62	−0.33
	DASS-Stress	2223	1.10	0.67	0.27	−0.60
OC group	ARCES	355	3.14	0.72	0.15	−0.31
	MAASLO	355	3.49	0.77	−0.02	0.34
	MWS	355	4.60	1.36	−0.43	−0.09
	MWD	355	4.58	1.47	−0.25	−0.60
	SBPS	355	3.42	1.14	0.32	−0.35
	DECE	355	4.11	1.22	−0.05	−0.48
	DECI	355	3.93	1.25	0.05	−0.42
	DASS-Dep	355	0.85	0.71	0.92	0.18
	DASS-Anx	355	0.87	0.66	0.68	−0.27
	DASS-Stress	355	1.16	0.64	0.17	−0.72

*Note*. ARCES = Attention Related and Cognitive Errors Scale, MAASLO = Mindful Attention Awareness Scale – Lapses Only, MWS = Mind Wandering Spontaneous scale, MWD = Mind Wandering Deliberate scale, SBPS = Boredom Proneness Scale – Short Form, DECE = Deep Effortless Concentration – External Scale, DECI = Deep Effortless Concentration – Internal Scale, DASS-Dep = DASS Depression Subscale, DASS-Anx = DASS Anxiety Subscale, DASS-Stress = DASS Stress Subscale.

**Table 3:** Cronbach alphas (left panel) and pearson correlations (right panel) of the OC group (above the diagonal; N = 355) and non-OC group (below the diagonal; N = 2223).

*Measure*	*OC Cronbach 𝛼*	*Non-OC Cronbach 𝛼*	*1*	*2*	*3*	*4*	*5*	*6*	*7*	*8*	*9*	*10*
1. MW-S	.89	.89	**--**	.45 **	.47 **	.60 **	.40 **	−.33 **	−.31 **	.28 **	.30 **	.34 **
2. MW-D	.92	.90	.44 **	**--**	.16 *	.29 **	.19 **	−.06	.01	.18 *	.17 *	.16 *
3. MAAS-LO	.87	.87	.47 **	.18 **	**--**	.58 **	.43 **	−.29 **	−.29 **	.32 **	.36 **	.42 **
4. ARCES	.91	.91	.50 **	.23 **	.56 **	**--**	.38 **	−.33 **	−.31 **	.31 **	.39 **	.44 **
5. SBPS	.87	.87	.42 **	.15 **	.51 **	.42 **	**--**	−.31 **	−.20 **	.60 **	.46 **	.42 **
6. DECE	.92	.96	−.26 **	−.03	−.25 **	−.29 **	−.35 **	**--**	.53 **	−.27 **	−.26 **	−.30 **
7. DECI	.96	.95	−.20 **	.07 *	−.21 **	−.29 **	−.27 **	.48 **	**--**	−.22 **	−.17 *	−.25 **
8.DASS-Depression	.95	.91	.32 **	.15 **	.43 **	.32 **	.56 **	−.21 **	−.14 **	**--**	.66 **	.70 **
9. DASS-Anxiety	.79	.81	.29 **	.14 **	.40 **	.37 **	.44 **	−.17 **	−.12 **	.72 **	**--**	.73 **
10. DASS-Stress	.83	.84	.34 **	.12 **	.45 **	.36 **	.47 **	−.19 **	−.15 **	.76 **	.77 **	**--**

*Note*. ARCES = Attention Related and Cognitive Errors Scale, MAASLO = Mindful Attention Awareness Scale – Lapses Only, MWS = Mind Wandering Spontaneous scale, MWD = Mind Wandering Deliberate scale, SBPS = Boredom Proneness Scale – Short Form, DECE = Deep Effortless Concentration – External Scale, DECI = Deep Effortless Concentration – Internal Scale, DASS-Dep = DASS Depression Subscale, DASS-Anx = DASS Anxiety Subscale, DASS-Stress = DASS Stress Subscale.

* p =.05, ** p <.001

### OC users vs. non-users

#### Comparing OC users who reported brand versus OC users who did not report brand.

Before examining whether OC formulation was related to differences in trait-level attention, we attempted to replicate our prior work comparing OC users and non-users [11,12]. However, given that not all OC users reported their brand, it was possible that OC users who reported their brand differed from those who did not report their brand. As such, we first sought to compare these two groups. To do this, we conducted seven planned comparisons (one on each of attention measures) comparing OC users who reported their brand (n = 255) to OC users who did not report their brand (n = 100). To control for multiple comparisons, we utilized a Bonferroni correction, setting alpha at.007 (.05/ 7). As can be seen in Fig 1, we found OC users who reported their brand did not significantly differ from OC users who did not report their brand on the ARCES, *t*(174.8) = 0.32, *p* = .749, *d* = .04, MAASLO, *t*(163.5) = 1.13, *p* = .259, *d* = .14, MWS, *t*(176.0) = 1.11, *p* = .267, *d* = .13, MWD, *t*(186.1) = 0.49, *p* = .621, *d* = .06, SBPS, *t*(165.4) = 0.73, *p* = .462, *d* = .09, DECE, *t*(181.5) = 1.27, *p* = .207, *d* = .15, and DECI, *t*(171.4) = 0.38, *p* = .704, *d* = .05.

**Fig 1:**
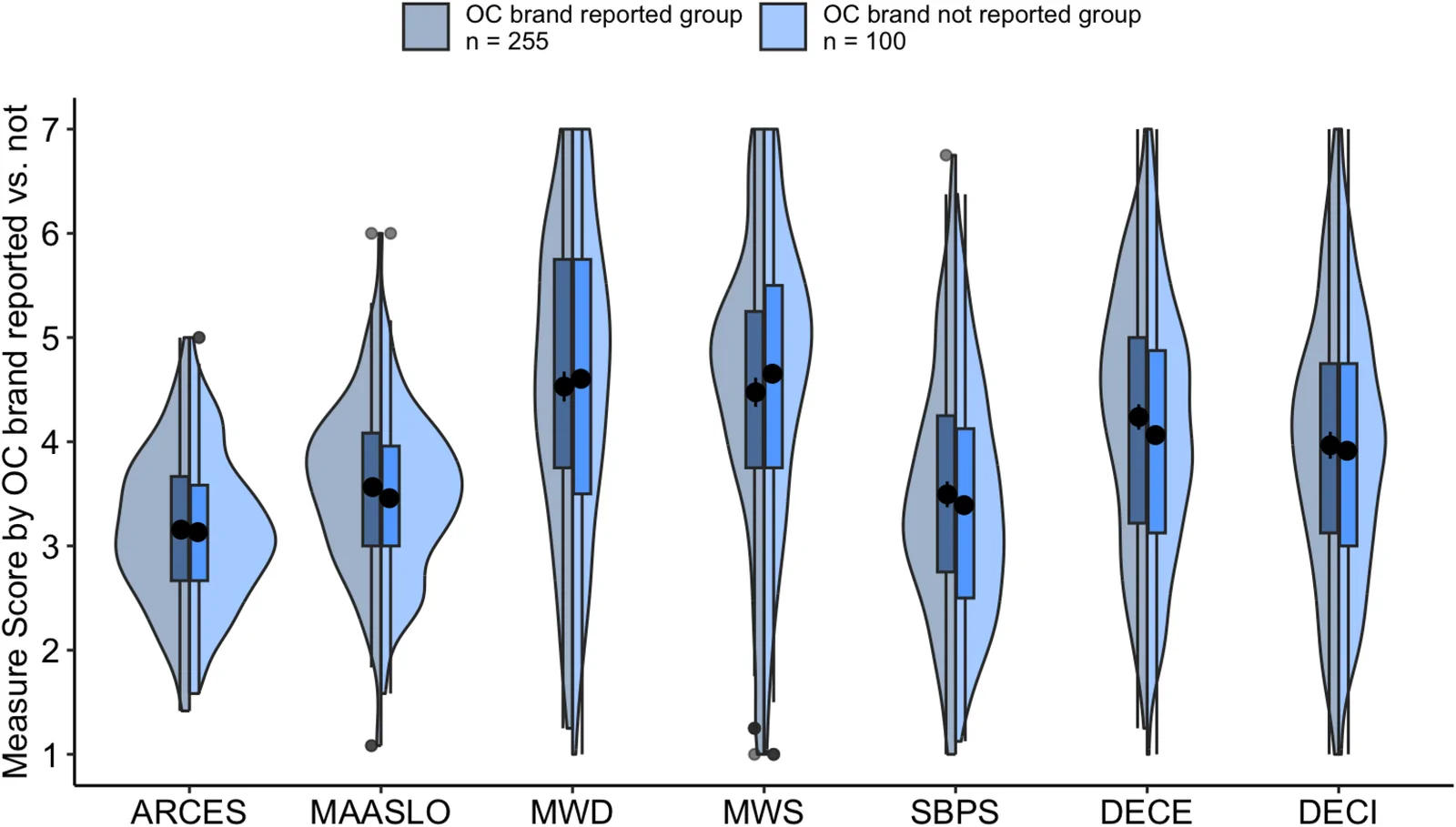
Split violin plots with box and whisker plots (boxplots) for each of the measures (ARCES, MAASLO, MWD, MWS, SBPS, DECE, and DECI) as a function of OC brand reported vs. not.

### OC use and Attention

Since we found no significant differences between OC users who reported their brand and those who did not report their brand, we collapsed across OC users to create one homogenous group of OC users that we could compare to non-users.

To do this, we conducted seven planned comparisons, comparing OC users and non-users on each of our attention measures of interest. To control for multiple comparisons, we utilized a Bonferroni correction, setting alpha at.007 (.05/ 7). We found no significant differences between groups on attention related errors, *t*(467.3) = 0.48, *p* = .630, *d* = .03, attention lapses, *t*(483.2) = 1.28, *p* = .199, *d* = .07, spontaneous mind wandering, *t*(464.2) = 0.53, *p* = .594, *d* = .03, deliberate mind wandering, *t*(466.7) = 1.78, *p* = .075, *d* = .10, boredom, *t*(478.0) = 2.68, *p* = .008, *d* = .15, internal flow, *t*(462.8) = 2.02, *p* = .044, *d* = .12, or external flow, *t*(479.1) = 0.44, *p* = .659, *d* = .02 (Fig 2). We should note that while not statistically significant using the Bonferroni correction, there was a numerical difference between groups in terms of boredom proneness that was significant at an uncorrected alpha, such that OC users had nominally less boredom proneness than non-users. These findings are consistent with our prior work [11,12]. This allows us to infer that the present findings are also applicable to those prior findings [11,12].

**Fig 2:**
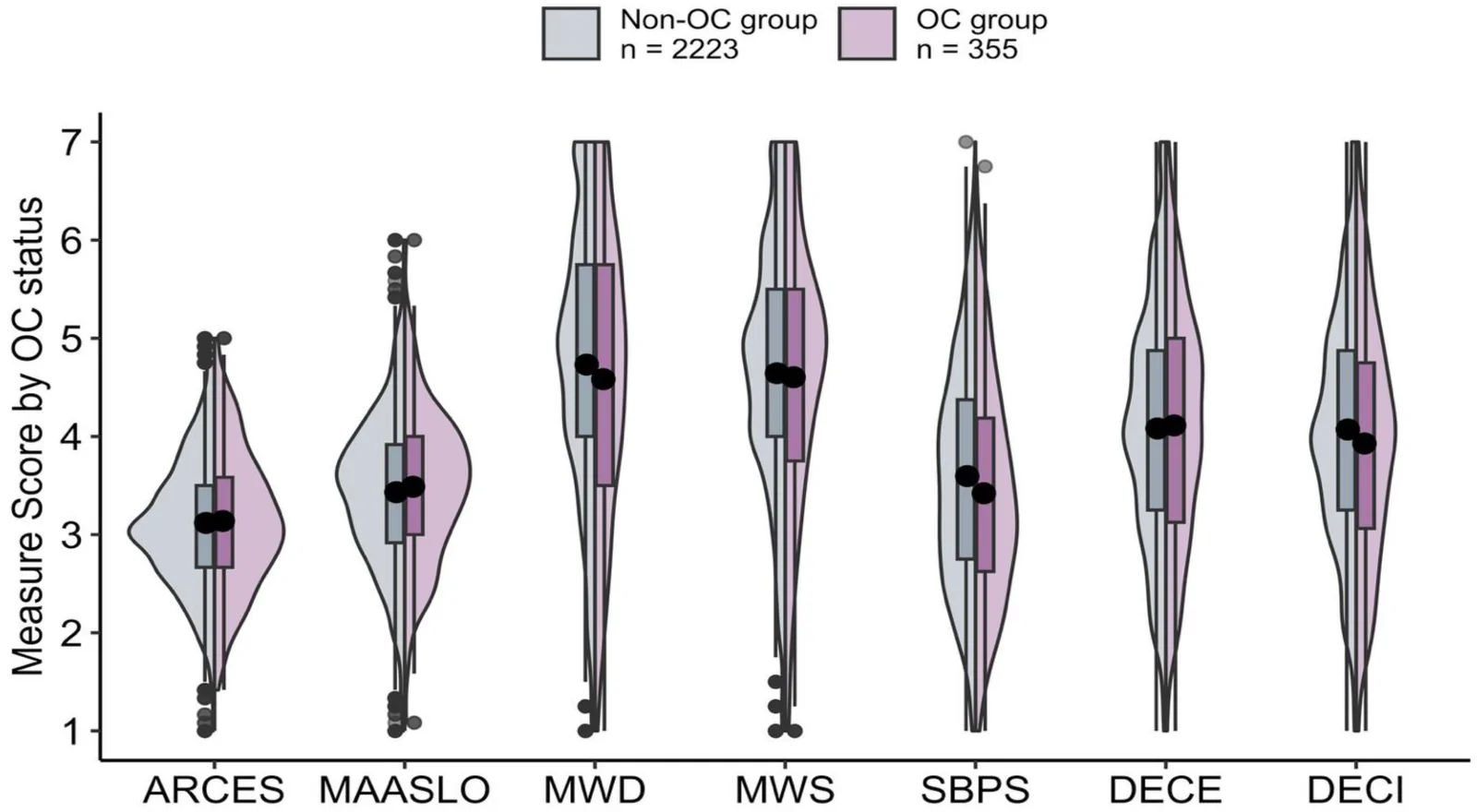
Split violin plots with box and whisker plots (boxplots) for each of the measures (ARCES, MAASLO, MWD, MWS, SBPS, DECE, and DECI) as a function of OC use or non-use.

### Describing OC composition

We provide the brand name, formulation and dose, dosing schedule, and hormonal activity for each OC participants reported using, as well as the number of participants who reported using that formulation in Table 4. Of the 355 OC users in our sample, 255 reported the brand they used (72%). We were able to identify the formulation for all 31 brands reported. Some formulations were branded under multiple names so although 31 different brands were reported, in total there were only 15 different formulations which varied by the ethinyl estradiol/ esterol monohydrate dose, progestin type, and/or progestin dose (Table 4).

**Table 4:** Oral contraceptives by dosing schedule, formulation and dose, and estrogenic, progestational, and androgenic activity (OC N = 355).

Brand name(s)	Dosing schedule	Formulation and dose	Progestin Generation	Dickey Profile No.	Estrogenic activity/ level	Progestational activity/ level	Androgenic activity/ level	N
Freya, Mirvala, Marvelon	Monophasic	Ethinyl estradiol 30 mcg/ Desogestrel 150 mcg	3	8	30/ intermediate high	1.5/ high	0.17/ intermediate low	20
Yasmin, Zamine	Monophasic	Ethinyl estradiol 30 mcg/ Drospirenone 3000 mcg	4	9	30/ intermediate high	1.5/ high	0/ low	8
Portia, Indayo, Seasonale, Seasonique, Microglynon 3, Ovima	Monophasic	Ethinyl estradiol 30 mcg/ Levonorgestrel 150 mcg	3	12	25/ intermediate low	0.8/ intermediate low	0.45/ intermediate high	10
Mya, Yaz, Yaz Plus	Monophasic	Ethinyl estradiol 20 mcg/ Drospirenone 3000 mcg	4	14	20/ intermediate low	1.5/ high	0/ low	23
Alysena, Alesse, Aviane	Monophasic	Ethinyl estradiol 20 mcg/ Levonorgestrel 100 mcg	3	15	17/ low	0.5/ intermediate low	0.31/ intermediate high	127
Lolo	Monophasic	Ethinyl estradiol 10 mcg/ Norethindrone Acetate 1000 mcg	1	18	12/ low	1.4/ high	0.64/ high	15
Diane-35, Teva-Cyproterone, Cleo 35	Monophasic	Ethinyl estradiol 35 mcg/ Cyproterone Acetate 2000 mcg	1	n/a	Could not categorize (formulation not in Dickey^48^)			4
Dienorette	Monophasic	Ethinyl estradiol 20 mcg/ Dienogest 30 mcg	4	n/a	Could not categorize (formulation not in Dickey^48^)			1
Nextstellis	Monophasic	Esterol Monohydrate 15000 mcg/ Drospirenone 3000 mcg	4	n/a	Could not categorize (formulation not in Dickey^48^)			2
Brevicon	Biphasic	Ethinyl estradiol 35 mcg/ Norethindrone 500, 1000 mcg	1	20	40/ high	0.8/ intermediate low	0.25/ intermediate high	1
Linessa	Triphasic	Ethinyl estradiol 25 mcg/ Desogestrel 100, 125, 150 mcg	3	25	25/ intermediate low	1.2/ intermediate high	0.14/ low	19
Tricira Lo	Triphasic	Ethinyl estradiol 25 mcg/ Norgestimate 180, 215, 250 mcg	3	26	25/ intermediate low	0.3/ low	0.15/ low	12
Tri-Jordyna 28	Triphasic	Ethinyl estradiol 35 mcg/ Norgestimate 180, 215, 250 mcg	3	31	35/ high	0.2/ low	0.15/ low	2
Movisse, Jencycla	Progestin Only	Norethindrone 250 mcg	1	33	0/ low	0.4/ low	0.13/ low	6
Slynd, Slinda	Progestin Only	Drospirenone 4000 mcg	4	n/a	Could not categorize (formulation not in Dickey^48^)			5
Not reported	Unknown	Unknown	Unknown	n/a	Unknown			100

**Note:** The estrogenic, progestational, and androgenic activity of OCs considers both the dose and the potency of the composite hormones. Activity values range on a continuous scale (with higher numbers indicating a greater potency; see Dickey [23]). These values have also been categorized into 4 levels (ranging from low to high; see Dickey [23]). Dickey profile number is provided as this was used to identify the estrogenic, progestational, and androgenic activity and level.

We use these different ways of categorizing OCs to analyze the relation between OC use and attention. Most OCs consist of a package of 28 pills which includes 21–24 days of ‘active’ pills containing ethinyl estradiol/ esterol monohydrate and/or progestin and 4–7 days of inactive or placebo pills (though some brands only contain the active pills and patients are instructed not to take a pill for 4–7 days before starting a new package). The majority of OC users in our sample reported using a monophasic OC (210/ 255; 82%; 206 included in analyses due to Nextstellis exclusions) in which patients receive a consistent dose of ethinyl estradiol and progestin in each their active pills. Fewer participants reported use of other dosing schedules (biphasic, triphasic, or progestin only). Only 1 (1/ 255; 0.39%) participant reported use of a biphasic OC, in which the dose of ethinyl estradiol is consistent but the dose of progestin increases once in the pack. Thirty-three OC users reported use of a triphasic OC (33/ 255; 13%), in which patients receive a consistent dose of ethinyl estradiol across their active pills but an increasing dose of progestin each week (i.e., dose of progestin changes twice in a pack). A small number of OC users also reported using progestin only OCs (11/ 255; 4%; percentages do not sum to 100 due to rounding) which contain no ethinyl estradiol, only a progestin (Table 4).

Within these different dosing schedules, OCs can vary further by other aspects of their formulation. While most monophasic and triphasic OCs are composed of both ethinyl estradiol (one monophasic OC formulation (brand name: Nextstellis) has an estradiol component of esterol monohydrate. Esterol is less potent and has a different binding affinity than ethinyl estradiol [34]. As a result, the dose of esterol monohydrate is much higher than the doses of ethinyl estradiol. Moreover these differences in binding affinity also lead to different effects on different tissues [34]. Together, this suggests this OC formulation in particular may act differently than the others and the large esterol monohydrate dose makes it difficult to compare with OCs composed of ethinyl estradiol. As such, we opted to exclude those (N = 4) who used this formulation from our analyses.) and progestin, ethinyl estradiol can vary by dose and estrogenic activity and the progestin can vary by type, dose, progestational activity, and androgenic activity (since some progestins are synthesized from testosterone). Progestin only OCs are composed of only progestin and can vary by the type, dose, progestational activity, and androgenic activity of the progestin. As we note earlier, estrogenic, progestational, and androgenic activities range from low to high (Table 4). Four formulations (used by 12 participants) of OCs were not included in Dickey [23] and as such, we could not determine the activity levels for those OCs. The participants without these activity levels are omitted from analyses by activity level.

We also assigned a generation for each progestin type. Most OCs in our sample were synthesized with a third generation progestin (190/ 255; 75%), while only 26 participants reported using an OC with a first generation progestin (10%), no participants reported using an OC with a second generation progestin (0%), and 39 participants using an OC containing a fourth generation progestin (15%; percentages do not sum to 100 due to rounding).

We also examined how the estrogenic, progestational, and androgenic activity of OCs related to our attention measures. The benefit of examining this activity (rather than just dose) is that it takes into account both the dose and the potency of the composite hormones. Activity values range on a continuous scale (with higher numbers indicating a greater potency; see Dickey [23]. These values have also been categorized into 4 levels (ranging from low to high; see Dickey [23]. Since little work has been conducted using these activity levels (to our knowledge Beltz et al [31] was the first to conduct such an analysis) we planned to analyze our data both using the continuous activity values in regression analyses predicting each attention measure of interest as well as using the activity level categories (4 levels; low to high; as per Beltz et al [31] as predictors in separate regression analyses of each attention measure. However, collapsing activity levels into categories created multicollinearity between two levels of activity (intermediate high androgenicity and intermediate low progesticity), which violated the independence assumption of regression. As such, we only include the regression analyses for the continuous activity levels.

In the following analyses, we investigate whether these different aspects of OC formulation are associated with differences in the tendency to experience boredom, attention related errors, attention lapses, spontaneous and deliberate mind wandering, and internal and external flow (or deep effortless concentration). We also sought to determine whether OC users who reported their brand differed from those who did not report their brand and replicate our prior work collapsing across OC formulations showing no significant differences in attention related errors, attention lapses, spontaneous and deliberate mind wandering, and internal and external flow (or deep effortless concentration [11,12], and significant differences in boredom proneness [12].

### OC Composition and Attention

#### Correlating Ethinyl-Estradiol Dose and Attention.

To determine whether ethinyl-estradiol (EE) dose was related to differences in attention, we conducted seven spearman correlations. One correlation between EE and each attention measure (ARCES, MAASLO, MWS, MWD, SBPS, DECE, and DECI). We included all OC users who reported their brand with the exception of the 4 participants using the OC brand Nextstellis (N = 251) as the estradiol component (esterol monohydrate). Since esterol is less potent and has a different binding affinity than ethinyl estradiol [34], the dose of esterol monohydrate is much higher than the doses of ethinyl estradiol. These participants were excluded from the present analyses as these doses were outliers. All dosing schedules were included (including participants using progestin only OCs because they represented a true zero in terms of EE dose). Spearman correlations were chosen because the distribution of EE doses was not normal. There were no significant correlations between EE dose and any of our attention measures (Table 5). When correlations were computed with only monophasic OC users included (N = 210), there was a nominal correlation between EE dose and external flow (as measured by the DECE) *r*(208) =.13, *p* = .054, while all other correlations did not reach significance, *r*s < .09, all *p*s ≥ .218. We lacked adequate power to compute meaningful (and stable) correlations within the triphasic or progestin only groups (n’s < 35; Schönbrodt & Perugini [35] suggest that correlations only stabilize at n ~ 250).

**Table 5:** Spearman correlations of trait-level attentional engagement, ethinyl-estradiol dose, and progestin dose.

Measure	*r* with EE dose	*r* with P dose
ARCES	*r*(251) = -.03, *p* =.587	*r*(219) =.02, *p* =.766
MAASLO	*r*(251) = -.04, *p* =.568	*r*(219) =.02, *p* =.803
MWS	*r*(251) =.01, *p* =.886	*r*(219) =.04, *p* =.587
MWD	*r*(251) = -.02, *p* =.775	*r*(219) =.05, *p* =.486
SBPS	*r*(251) =.06, *p* =.333	*r*(219) =.06, *p* =.410
DECE	*r*(251) =.08, *p* =.204	*r*(219) = -.03, *p* =.685
DECI	*r*(251) = -.03, *p* =.661	*r*(219) = -.01, *p* =.850

*Note*. ARCES = Attention Related and Cognitive Errors Scale, MAASLO = Mindful Attention Awareness Scale – Lapses Only, MWS = Mind Wandering Spontaneous scale, MWD = Mind Wandering Deliberate scale, SBPS = Boredom Proneness Scale – Short Form, DECE = Deep Effortless Concentration – External Scale, DECI = Deep Effortless Concentration – Internal Scale; EE = Ethinyl-Estradiol; P = Progestin

### Correlating progestin dose and attention

We also investigated whether progestin dose was related to differences in attention by again computing seven Spearman correlations between progestin dose (which was non-normally distributed) and each of the attention measures. Since biphasic and triphasic OC users receive 2–3 different doses of progestin in their pack, we excluded these participants (N = 34) from this analysis as it was unclear which dose to include in this correlation. As such, these correlations include monophasic and progestin only OC users (N = 221). We found no significant correlations between progestin dose and reports of attention (Table 5). When examining only monophasic OC users, these correlations remained non-significant all *r*s < .10, all *p*s ≥ .410. Again, since the triphasic and progestin only groups were small, we opted not to calculate these correlations with those groups individually.

### OC activity and attention

To investigate the role of (continuous) activity levels we regressed estrogenic activity, progestational activity, and androgenic activity on each of our attention measures of interest. Activity did not predict a significant amount of variance in any of our attention measures (Table 6). When predicting boredom, progestational activity was a significant positive predictor of boredom (measured via the SBPS), such that more progestational activity was related to more boredom proneness. No other activities were significant predictors of our attention measures.

**Table 6:** Regression models using continuous hormonal activity values to predict each attention measure.

	Beta	*SE*	*p*
**DV: ARCES**	**R**^**2**^ **= .00, F(3,233) = 0.29, SE = 0.71, Model *p* = .834**		
Intercept	3.14	0.05	<.001
Est. Activity	−0.01	0.01	.457
Prog. Activity	0.09	0.11	.436
And. Activity	0.04	0.34	.915
**DV: MAASLO**	**R**^**2**^ **= .01, F(3,233) = 1.00, SE = 0.75, Model *p* = .395**		
Intercept	3.47	0.05	<.001
Est. Activity	−0.00	0.01	.710
Prog. Activity	0.21	0.12	.088
And. Activity	0.08	0.35	.828
**DV: MWS**	**R**^**2**^ **= .01, F(3,233) = 0.42, SE = 1.35, Model *p* = .742**		
Intercept	4.68	0.09	<.001
Est. Activity	−0.01	0.02	.720
Prog. Activity	0.24	0.22	.269
And. Activity	0.01	0.64	.993
**DV: MWD**	**R**^**2**^ **= .01, F(3,233) = 0.88, SE = 1.47, Model *p* = .454**		
Intercept	4.55	0.10	<.001
Est. Activity	−0.03	0.02	.115
Prog. Activity	0.13	0.24	.587
And. Activity	−0.16	0.70	.814
**DV: SBPS**	**R**^**2**^ **= .03, F(3,233) = 3.04, SE = 1.09, Model *p* = .030**		
Intercept	3.44	0.07	<.001
Est. Activity	−0.01	0.01	.494
Prog. Activity	0.47	0.18	.008 *
And. Activity	−0.46	0.52	.378
**DV: DECE**	**R**^**2**^ **= .01, F(3,233) = 1.09, SE = 1.22, Model *p* = .353**		
Intercept	4.04	0.08	<.001
Est. Activity	0.01	0.02	.488
Prog. Activity	−0.23	0.20	.243
And. Activity	−0.70	0.58	.224
**DV: DECI**	**R**^**2**^ **= .00, F(3,233) = 0.24, SE = 1.22, Model *p* = .869**		
Intercept	3.87	0.08	<.001
Est. Activity	−0.00	0.02	.974
Prog. Activity	−0.08	0.20	.679
And. Activity	0.30	0.58	.601

*Note 1*: DV = Dependent variable; ARCES = Attention Related Errors Scale, MAASLO = Mindful Attention Awareness Scale – Lapses Only, MWD = Mind Wandering Deliberate scale, MWS = Mind Wandering Spontaneous scale, SBPS = Boredom Proneness Scale – Short Form, DECE = Deep Effortless Concentration – External Scale, DECI = Deep Effortless Concentration – Internal Scale, Est. Activity = Estrogenic Activity, Prog. Activity = Progestational Activity, And. Activity = Androgenic Activity

*Note 2:* Activity values were standardized and centered prior to regression analyses.

### Dosing schedule

We conducted seven one-way analyses of variance (ANOVA) to determine whether differences in dosing schedules predicted our attention measures. We entered dosing schedule (monophasic, triphasic or progestin only) as a between participants factor and conducted an ANOVA with each attention measure as the dependent variable (Fig 3). Given that we only had one participant report using a biphasic OC, this participant was omitted from the following analyses. We also only had 33 triphasic OC users and 11 progestin only OC users, so we were under-powered to detect differences between these groups. As such, these results should be interpreted with caution. There was no significant main effect of dosing schedule on attention related errors (as indexed by the ARCES), *F*(3, 248) = 0.74, MSE = 0.50, *p* = .529, η_p_^2^ = 0.01, attention lapses (as indexed by the MAASLO), *F*(3, 248) = 0.39, MSE = 0.56, *p* = .761, η_p_^2^ = 0.01, spontaneous mind wandering (as indexed by the MWS), *F*(3, 248) = 1.30, MSE = 1.82, *p* = .276, η_p_^2^ = 0.02, deliberate mind wandering (as indexed by the MWD), *F*(3, 248) = 1.60, MSE = 2.16, *p* = .190, η_p_^2^ = 0.02, boredom proneness (as indexed by the SBPS), *F*(3, 248) = 0.66, MSE = 1.23, *p* = .577, η_p_^2^ = 0.01, internal flow (as indexed by the DECI), *F*(3, 248) = 0.84, MSE = 1.48, *p* = .471, η_p_^2^ = 0.01, or external flow (as indexed by the DECE), *F*(3, 248) = 2.25, MSE = 1.45, *p* = .083, η_p_^2^ = 0.03.

**Fig 3:**
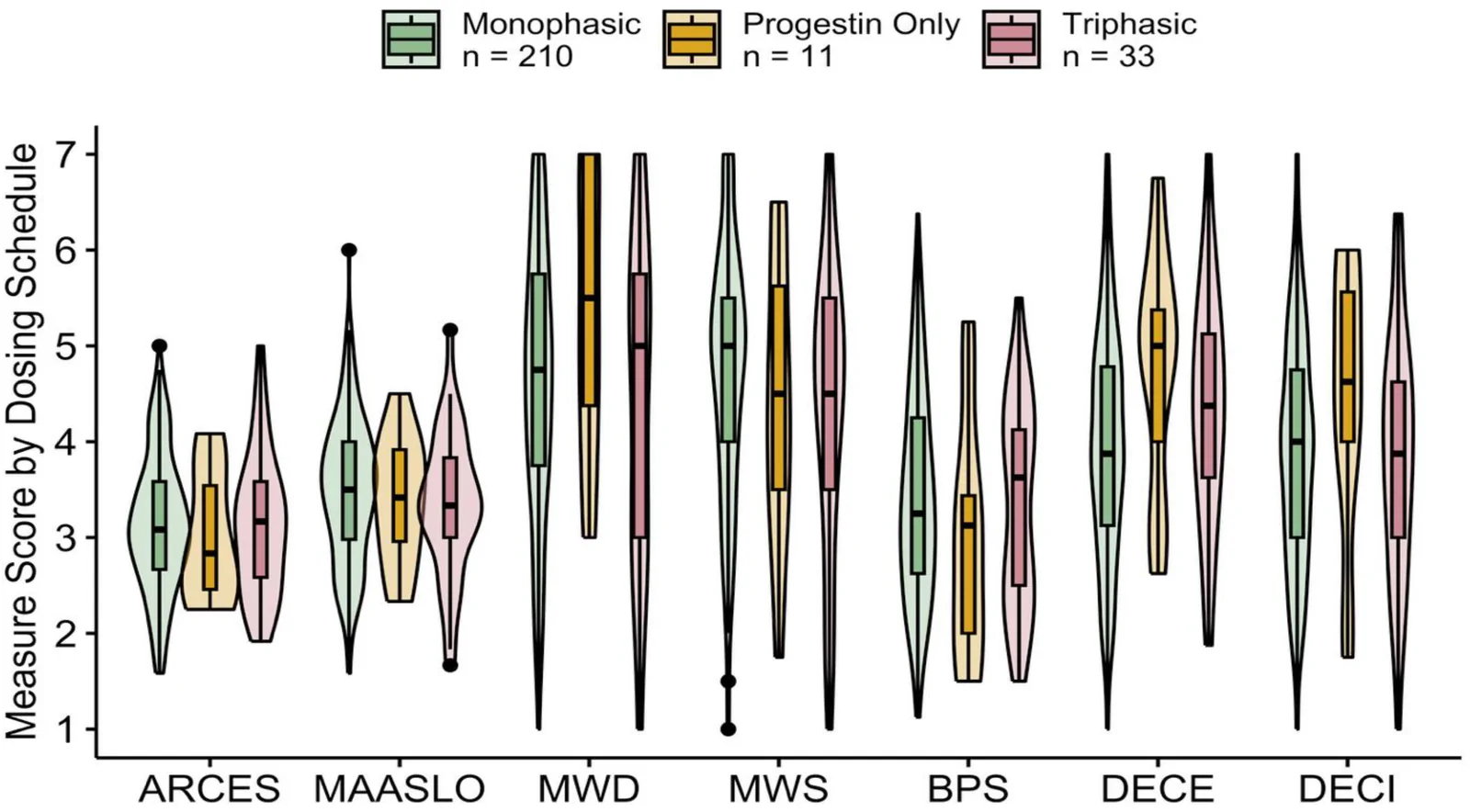
Violin plots with box and whisker plots (boxplots) for each of the measures (ARCES, MAASLO, MWD, MWS, SBPS, DECE, and DECI) as a function of OC dosing schedule. Note: Biphasic OC users (n = 1) are not included in the figure.

### Generation

Finally, to determine whether progestin generation predicted the attention measures we conducted seven one-way ANOVAs entering progestin generation as a between participants factor and each attention measure as the dependent variable in a separate ANOVA. We note that we are likely under-powered to detect small or medium sized differences in those using a first or fourth generation progestin since these groups at quite small (ns ≤ 26). We also lack data on second generation progestins since none of our participants reported use of OCs with this type of progestin. As such, the following results should be considered to be preliminary.

There was no significant main effect of generation on attention related errors, *F*(2, 249) = 0.50, MSE = 0.51, *p* = .608, η_p_^2^ = 0.00, attention lapses, *F*(2, 249) = 0.03, MSE = 0.56, *p* = .968, η_p_^2^ = 0.00, spontaneous mind wandering, *F*(2, 249) = 0.40, MSE = 1.84, *p* = .671, η_p_^2^ = 0.00, deliberate mind wandering, *F*(2, 249) = 1.24, MSE = 2.17, *p* = .291, η_p_^2^ = 0.01, boredom proneness, *F*(2, 249) = 0.07, MSE = 1.24, *p* = .936, η_p_^2^ = 0.00, external flow, *F*(2, 249) = 1.60, MSE = 1.47, *p* = .203, η_p_^2^ = 0.01, or internal flow, *F*(2, 249) = 0.13, MSE = 1.48, *p* = .878, η_p_^2^ = 0.00 (Fig 4).

**Fig 4:**
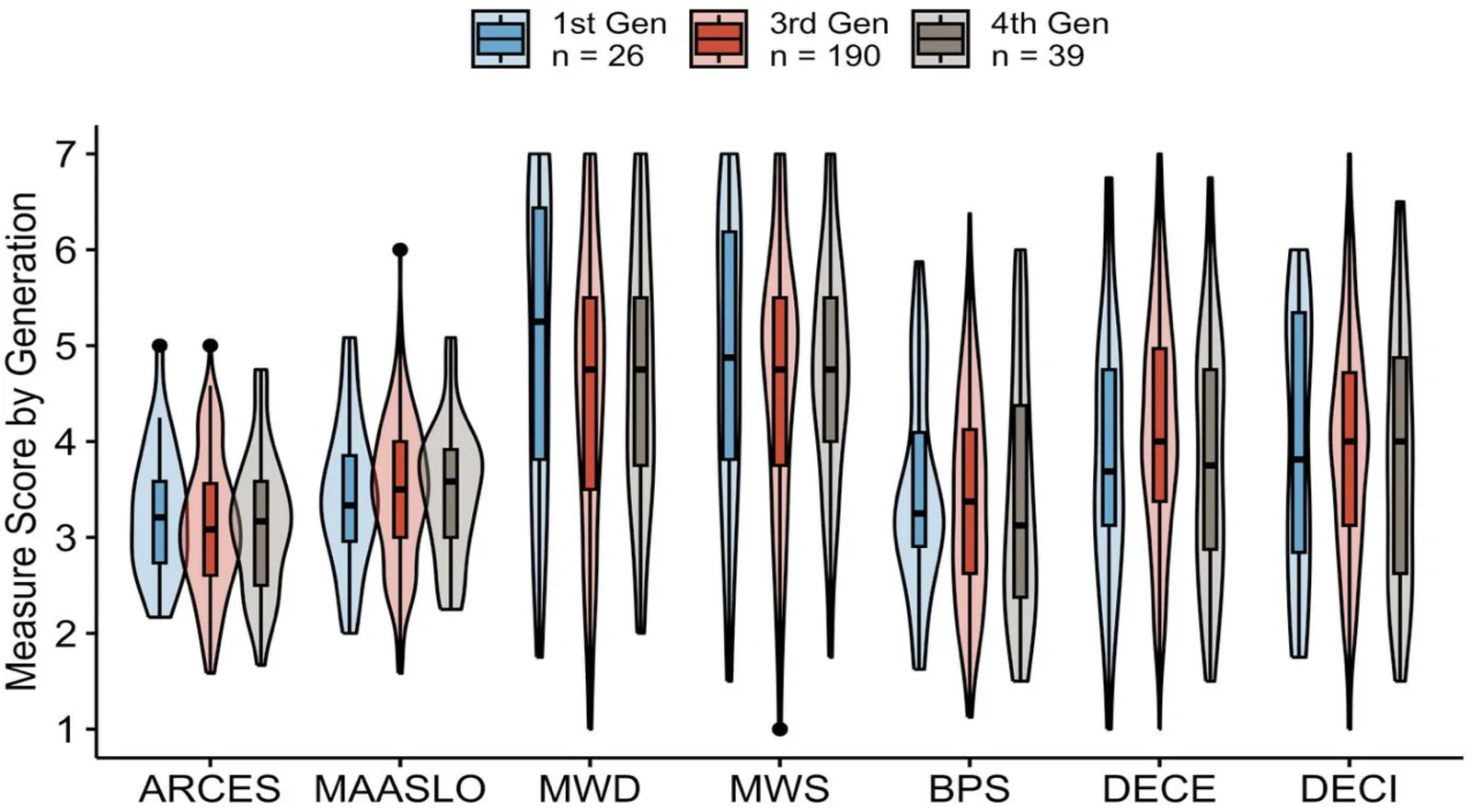
Violin plots with box and whisker plots (boxplots) for each of the measures (ARCES, MAASLO, MWD, MWS, SBPS, DECE, and DECI) as a function of OC generation (Gen).

## Discussion

In the present investigation, we aimed to address a limitation in our prior work investigating OC use and attention-related traits by considering the role of OC formulation in these relations. Specifically, we explored whether different aspects of OC formulation were related to differences in everyday attentional engagement as well as the propensity to experience boredom and internal and external flow. Our findings indicated that none of the OC formulation aspects (ethinyl-estradiol dose, progestin dose, hormonal activity, dosing schedule, progestin generation) were significantly related to differences in the traits measured. We also compared these same traits in OC users who provided the brand of OC used to OC users who did not provide the brand used and again found no significant differences between groups. This suggested that we could collapse across OC formulation and consider all OC users as a homogenous group in an attempt to replicate our prior work [11,12] compare to OC users and non-users. This comparison largely replicated our previous work showing that OC users and non-users do not significantly differ in the tendency to experience spontaneous and deliberate mind wandering, attention related errors, attention lapses, and internal and external flow [11,12]. In the present study, we found that OC users reported lower boredom proneness than non-users as we did in our prior work [12], although this difference was only statistically significant before we corrected for multiple comparisons. Importantly, given the present findings we are confident that collapsing across OC formulation in our present (and prior) work is not obscuring differences that emerge when OC formulation is accounted for.

We hasten to add that our non-significant differences in attention-related experiences across OC formulation could be the result of a lack of power. Many of our analyses required us to split the OC group into several subgroups. That is, while we have a large group of OC users who reported their brand overall (N = 255), giving us sufficient power to detect medium and small sized effects when looking at aspects of OC formulation on a continuous scale (i.e., ethinyl-estradiol dose, progestin dose, hormonal activity), we lost power when we split this group by dosing schedule and generation. When looking at dosing schedule, the vast majority of our participants used a monophasic OC (other work has also shown that most OC users use a monophasic OC [36]; These results make sense in the context of prescription guidelines for physicians which recommend prescribing a monophasic OC first. The consistent hormone dose is easier to adapt to, and more is known about the pharmacological action and side effects since monophasic OCs have been around longer than multiphasic formulations. There is also some evidence monophasic OCs are better tolerated in terms of side effects [37–40].. Unfortunately, our sample sizes in the progestin only and biphasic groups were not even powered to detect large sized effects (large sized effects require an N ~ 25 per group [41]). We were also underpowered to detect medium and small sized effects between the triphasic, progestin only, and biphasic groups (medium effects require N ~ 62 per group, small effects require N > 80–100 [41]). A similar power issue emerged when examining progestin generation. Again, here most participants used a third generation progestin, so we were underpowered to detect medium or small sized effects when comparing to the first or fourth generation groups (it is difficult to say whether our sample is representative of progestin generation among OC users as to our knowledge there is little to no work on progestin generation prevalence). Though we do not find significant differences in everyday attention by dosing schedule or generation, it possible that in a larger sample with more power differences may emerge. However, we wish to emphasize that the goal of this study was not to determine whether OC formulation was related to differences in attention (for that a much larger sample is required) but to explore whether it was possible that the results of our prior work were obscuring differences in attention that would emerge only when formulation was accounted for. We find that this is not the case.

That said, it is also possible that our results do reflect the true state of OC use and everyday attentional engagement – with the exception of boredom proneness – in that, OC use is just not related to differences in attention-related traits. There are several observations that are consistent with this conclusion. First, a broad examination of the literature reveals that sex differences in cognitive performance [42,43] are only found in particular cognitive domains. Only three cognition domains have shown some sex differences: spatial tasks such as mental rotation [44,45], mathematical performance where sex differences tend to be small (or non-existent) but there tend to be more high performing boys than girls [46–49], and verbal abilities where girls tend to outperform boys [42]. Some researchers have noted that while some small differences in cognition exist between sexes, there is much more overlap between the sexes than there are differences [43]. These prior sex-related findings are consistent with small to null effects of hormonal variations in OCs on attentional experiences. Second, while some work does show that OCs alter brain structure and function, this work is often inconsistent. For example, some work shows that OC use is associated with increased connectivity in the Default Mode Network (DMN) – the brain network associated with attentional disengagement – while other work shows decreased connectivity or no differences in connectivity [50–52]. The lack of consistent findings with respect to the DMN may suggest there are no true neural differences in the connectivity of this network between OC users and non-users, which would fit with the null effects reported here and in our prior work. Finally, it is important to consider the role of the relation between attention and mood. In this regard, we have previously observed that differences in boredom proneness [12] between OC uers and non-users become non-significant once we symptoms of depression are accounted for. This suggests that group differences in boredom might be driven by differences in mood – not differences in cognition – emphasizing the importance of controlling for third variables known to vary with both OC use and cognition.

This work suggests several future directions. First, this study included a sample of undergraduates who reported their sex as female. Since undergraduates can be a homogenous sample in terms of age, education, and socioeconomic background, it is possible that our sample is not representative of OC users more generally [53,54]. Future work could utilize platforms that recruit a more heterogenous sample (e.g., Prolific) and examine how the results compare to those of the present study. Second, we did not account for several possible confounding factors. While we would have preferred to control for variables that are known to vary with OC use (e.g., depression, duration of OC use), we opted not to as once we split the data by OC formulation many groups were already underpowered and controlling for additional factors would have decreased the power further. Future work could recruit larger samples in order to be able to control for possible confounding factors without compromising power. We provide suggestions for recruiting these large samples in Smith, Smilek, and Fenske (under review) [55]. Third, as we have noted earlier, the goal of the present study was to determine whether collapsing across OC formulation obscured differences in everyday attention that would emerge once formulation was included. The goal was not to determine whether differences in OC formulation were related to differences in everyday attention. The latter question would require a much larger sample in order adequately power groups once the sample was split by the different aspects of OC formulation (e.g., dosing schedule, progestin generation). Nevertheless, this is a question worthy of investigation and could be examined in future work.

It is important to note that there are aspects of studying OCs which make it enharently difficult to elucidate the true effects of OC’s on cognition. The first factor is the survivor effect: prior work has shown that when individuals start using an OC and experience an adverse effect (like depression) they are likely to discontinue use after a short period of time [56–58]. Since these individuals are only on an OC for a short period of time, they are unlikely to have their experience captured in a study that recruits from a community sample (such as this one). The increased risk of depression among OC users tends to emerge in studies that are able to account for participants who use an OC for a short period of time (e.g., Skovlund et al [17] which utilized Denmark’s National Prescription Register and Psychiatric Central Research Register). It also possible that there are effects of OC use on cognition (e.g., attention-related experiences) shortly after participants first begin to use OCs and that these effects progressively disappear with time as users habituate to the medication. Indeed, it has been argued that some side effects of OCs will decrease or diminish after the second or third cycle of use as the body habituates to the lower endogenous hormone level and consistent exogenous hormone level [23](p28). It is possible that an individual may notice they struggle to focus during the first two cycles of a new OC, but their assessment of their side effects may change over time. For example, when first beginning an OC an individual might notice they are struggling to focus but this could be re-evaluated after longer use as ‘normal’. To tackle these issues, it may be necessary to conduct more longitudinal studies assessing cognition on a finer time scale following OC users over time (e.g., measuring attention on a daily or bi-daily basis over a 3–6 month period following commencement of an OC regimen).

## Supporting information

S1 Table Descriptive statistics of measures by semester and OC status. (DOCX)
