# Supplementary material for: Revisiting the relation between oral contraceptive use and attentional engagement in everyday life: The role of formulation
Source: PLoS One. 2026 Oct 6;21(10):e0359652. doi: 10.1371/journal.pone.0359652 (PMC13641381; doi:10.1371/journal.pone.0359652)
Supplement: Supplementary file 1 [file pone.0359652.s001.docx]

**Revisiting the relation between oral contraceptive use and attentional engagement in everyday life: The role of formulation**

**Supplementary Materials**

**Table S1.** Descriptive statistics of measures by semester and OC status

|  | **Semester** | | **Group** | | **Measure** | | **N** | **Mean** | | **SD** | | **Skew** | **Kurtosis** |
| --- | --- | --- | --- | --- | --- | --- | --- | --- | --- | --- | --- | --- | --- |
|  | Fall 2023 | | Non-OC group | | ARCES | | 745 | 3.13 | | 0.73 | | 0.19 | 0.20 |
|  |  | |  | | MAASLO | | 745 | 3.42 | | 0.81 | | -0.19 | 0.37 |
|  |  | |  | | MWS | | 745 | 4.62 | | 1.36 | | -0.32 | -0.21 |
|  |  | |  | | MWD | | 745 | 4.76 | | 1.45 | | -0.38 | -0.41 |
|  |  | |  | | SBPS | | 745 | 3.58 | | 1.23 | | 0.20 | -0.60 |
|  |  | |  | | DECE | | 745 | 4.10 | | 1.27 | | 0.06 | -0.37 |
|  |  | |  | | DECI | | 745 | 4.09 | | 1.17 | | 0.10 | -0.32 |
|  |  | |  | | DASS-Dep | | 745 | 0.99 | | 0.80 | | 0.66 | -0.47 |
|  |  | |  | | DASS-Anx | | 745 | 0.93 | | 0.73 | | 0.61 | -0.43 |
|  |  | |  | | DASS-Stress | | 745 | 1.13 | | 0.70 | | 0.43 | -0.40 |
|  |  | | OC group | | ARCES | | 151 | 3.12 | | 0.76 | | 0.12 | −0.46 |
|  |  | |  | | MAASLO | | 151 | 3.49 | | 0.80 | | 0.02 | -0.62 |
|  |  | |  | | MWS | | 151 | 4.57 | | 1.37 | | -0.53 | -0.14 |
|  |  | |  | | MWD | | 151 | 4.55 | | 1.51 | | -0.17 | -0.83 |
|  |  | |  | | SBPS | | 151 | 3.50 | | 1.20 | | 0.27 | -0.40 |
|  |  | |  | | DECE | | 151 | 4.16 | | 1.22 | | -0.16 | -0.47 |
|  |  | |  | | DECI | | 151 | 3.90 | | 1.23 | | 0.04 | -0.50 |
|  |  | |  | | DASS-Dep | | 151 | 0.86 | | 0.75 | | 0.92 | 0.04 |
|  |  | |  | | DASS-Anx | | 151 | 0.82 | | 0.69 | | 0.88 | 0.13 |
|  |  | |  | | DASS-Stress | | 151 | 1.08 | | 0.65 | | 0.43 | -0.40 |
|  | Winter 2024 | | Non-OC group | | ARCES | | 460 | 3.06 | | 0.67 | | 0.06 | 0.24 |
|  |  | |  | | MAASLO | | 460 | 3.42 | | 0.83 | | -0.03 | 0.53 |
|  |  | |  | | MWS | | 460 | 4.59 | | 1.25 | | -0.31 | -0.10 |
|  |  | |  | | MWD | | 460 | 4.71 | | 1.40 | | -0.29 | -0.30 |
|  |  | |  | | SBPS | | 460 | 3.53 | | 1.13 | | 0.22 | -0.35 |
|  |  | |  | | DECE | | 460 | 4.16 | | 1.15 | | 0.11 | -0.19 |
|  |  | |  | | DECI | | 460 | 4.17 | | 1.17 | | 0.08 | -0.17 |
|  |  | |  | | DASS-Dep | | 460 | 0.96 | | 0.75 | | 0.58 | -0.62 |
|  |  | |  | | DASS-Anx | | 460 | 0.91 | | 0.67 | | 0.52 | -0.48 |
|  |  | |  | | DASS-Stress | | 460 | 1.06 | | 0.67 | | 0.23 | -0.66 |
|  |  | | OC group | | ARCES | | 52 | 3.07 | | 0.67 | | 0.09 | -0.05 |
|  |  | |  | | MAASLO | | 52 | 3.50 | | 0.79 | | -0.38 | 3.74 |
|  |  | |  | | MWS | | 52 | 4.62 | | 1.45 | | -0.55 | 0.58 |
|  |  | |  | | MWD | | 52 | 4.76 | | 1.52 | | -0.37 | -0.31 |
|  |  | |  | | SBPS | | 52 | 3.25 | | 1.07 | | 0.24 | -0.46 |
|  |  | |  | | DECE | | 52 | 4.14 | | 1.16 | | 0.17 | -1.00 |
|  |  | |  | | DECI | | 52 | 4.18 | | 1.22 | | 0.05 | -0.16 |
|  |  | |  | | DASS-Dep | | 52 | 0.78 | | 0.63 | | 0.77 | -0.06 |
|  |  | |  | | DASS-Anx | | 52 | 0.92 | | 0.69 | | 0.42 | -1.12 |
|  |  | |  | | DASS-Stress | | 52 | 1.16 | | 0.60 | | 0.11 | -0.77 |
|  | Spring 2024 | | Non-OC group | | ARCES | | 104 | 3.08 | | 0.67 | | 0.41 | 0.34 |
|  |  | |  | | MAASLO | | 104 | 3.43 | | 0.75 | | 0.18 | 0.39 |
|  |  | |  | | MWS | | 104 | 4.62 | | 1.29 | | -0.26 | -0.12 |
|  |  | |  | | MWD | | 104 | 4.57 | | 1.40 | | -0.46 | -0.22 |
|  |  | |  | | SBPS | | 104 | 3.71 | | 1.21 | | 0.22 | -0.52 |
|  |  | |  | | DECE | | 104 | 4.06 | | 1.28 | | -0.03 | -0.68 |
|  |  | |  | | DECI | | 104 | 4.06 | | 1.21 | | 0.15 | -0.31 |
|  |  | |  | | DASS-Dep | | 104 | 0.93 | | 0.81 | | 0.83 | -0.31 |
|  |  | |  | | DASS-Anx | | 104 | 0.79 | | 0.69 | | 0.87 | 0.06 |
|  |  | |  | | DASS-Stress | | 104 | 1.04 | | 0.71 | | 0.32 | -0.84 |
|  |  | | OC group | | ARCES | | 9 | 3.03 | | 0.86 | | 0.50 | -0.92 |
|  |  | |  | | MAASLO | | 9 | 3.31 | | 0.83 | | 0.21 | -1.00 |
|  |  | |  | | MWS | | 9 | 4.50 | | 1.45 | | -1.42 | 2.93 |
|  |  | |  | | MWD | | 9 | 3.72 | | 1.42 | | 0.00 | -2.00 |
|  |  | |  | | SBPS | | 9 | 2.44 | | 1.12 | | 1.29 | 0.83 |
|  |  | |  | | DECE | | 9 | 4.51 | | 1.42 | | 0.50 | -0.16 |
|  |  | |  | | DECI | | 9 | 3.93 | | 1.26 | | 0.08 | -0.56 |
|  |  | |  | | DASS-Dep | | 9 | 0.84 | | 0.68 | | 1.04 | 0.56 |
|  |  | |  | | DASS-Anx | | 9 | 0.90 | | 0.82 | | 1.24 | 1.17 |
|  |  | |  | | DASS-Stress | | 9 | 1.27 | | 0.79 | | 0.35 | -1.02 |
| Fall 2024 | | | Non-OC group | | ARCES | | 447 | 3.18 | | 0.71 | | 0.31 | -0.02 |
|  | | |  | | MAASLO | | 447 | 3.43 | | 0.76 | | -0.08 | 0.28 |
|  | | |  | | MWS | | 447 | 4.72 | | 1.31 | | -0.31 | -0.01 |
|  | | |  | | MWD | | 447 | 4.78 | | 1.42 | | -0.45 | -0.03 |
|  | | |  | | SBPS | | 447 | 3.62 | | 1.13 | | 0.18 | -0.28 |
|  | | |  | | DECE | | 447 | 3.99 | | 1.25 | | 0.10 | -0.21 |
|  | | |  | | DECI | | 447 | 3.96 | | 1.23 | | 0.10 | -0.09 |
|  | | |  | | DASS-Dep | | 447 | 0.92 | | 0.71 | | 0.65 | -0.48 |
|  | | |  | | DASS-Anx | | 447 | 0.93 | | 0.67 | | 0.63 | -0.21 |
|  | | |  | | DASS-Stress | | 447 | 1.13 | | 0.66 | | 0.20 | -0.67 |
|  | | | OC group | | ARCES | | 77 | 3.15 | | 0.71 | | 0.42 | 0.04 |
|  | | |  | | MAASLO | | 77 | 3.46 | | 0.70 | | -0.17 | -0.18 |
|  | | |  | | MWS | | 77 | 4.42 | | 1.38 | | -0.04 | -0.41 |
|  | | |  | | MWD | | 77 | 4.40 | | 1.37 | | -0.24 | -0.08 |
|  | | |  | | SBPS | | 77 | 3.37 | | 1.12 | | 0.43 | -0.27 |
|  | | |  | | DECE | | 77 | 4.06 | | 1.26 | | -0.12 | -0.35 |
|  | | |  | | DECI | | 77 | 3.92 | | 1.34 | | -0.10 | -0.52 |
|  | | |  | | DASS-Dep | | 77 | 0.90 | | 0.71 | | 0.74 | -0.20 |
|  | | |  | | DASS-Anx | | 77 | 0.92 | | 0.60 | | 0.48 | -0.37 |
|  | | |  | | DASS-Stress | | 77 | 1.23 | | 0.65 | | -0.06 | -0.62 |
| Winter 2025 | | Non-OC group | | ARCES | | 467 | | | 3.12 | | 0.66 | 0.25 | 0.42 |
|  | |  | | MAASLO | | 467 | | | 3.46 | | 0.78 | -0.12 | 0.06 |
|  | |  | | MWS | | 467 | | | 4.67 | | 1.28 | -0.48 | 0.17 |
|  | |  | | MWD | | 467 | | | 4.70 | | 1.41 | -0.35 | -0.34 |
|  | |  | | SBPS | | 467 | | | 3.64 | | 1.10 | 0.05 | -0.56 |
|  | |  | | DECE | | 467 | | | 4.06 | | 1.26 | 0.03 | -0.38 |
|  | |  | | DECI | | 467 | | | 4.06 | | 1.19 | 0.17 | -0.18 |
|  | |  | | DASS-Dep | | 467 | | | 0.93 | | 0.71 | 0.66 | -0.40 |
|  | |  | | DASS-Anx | | 467 | | | 0.88 | | 0.66 | 0.64 | -0.22 |
|  | |  | | DASS-Stress | | 467 | | | 1.10 | | 0.64 | 0.29 | -0.39 |
|  | | OC group | | ARCES | | 66 | | | 3.24 | | 0.62 | -0.02 | -0.32 |
|  | |  | | MAASLO | | 66 | | | 3.54 | | 0.76 | 0.24 | 1.09 |
|  | |  | | MWS | | 66 | | | 4.88 | | 1.23 | -0.39 | -0.20 |
|  | |  | | MWD | | 66 | | | 4.85 | | 1.38 | -0.52 | -0.22 |
|  | |  | | SBPS | | 66 | | | 3.55 | | 1.03 | 0.44 | -0.11 |
|  | |  | | DECE | | 66 | | | 3.99 | | 1.20 | 0.06 | -0.30 |
|  | |  | | DECI | | 66 | | | 3.81 | | 1.20 | 0.35 | 0.03 |
|  | |  | | DASS-Dep | | 66 | | | 0.85 | | 0.67 | 1.23 | 1.34 |
|  | |  | | DASS-Anx | | 66 | | | 0.91 | | 0.63 | 0.61 | -0.29 |
|  | |  | | DASS-Stress | | 66 | | | 1.25 | | 0.62 | -0.17 | -1.12 |

*Note*. ARCES = Attention Related and Cognitive Errors Scale, MAASLO = Mindful Attention Awareness Scale – Lapses Only, MWS = Mind Wandering Spontaneous scale, MWD = Mind Wandering Deliberate scale, SBPS = Boredom Proneness Scale – Short Form, DECE = Deep Effortless Concentration – External Scale, DECI = Deep Effortless Concentration – Internal Scale, DASS-Dep = DASS Depression Subscale, DASS-Anx = DASS Anxiety Subscale, DASS-Stress = DASS Stress Subscale.
