# Supplementary material for: Revisiting the relation between oral contraceptive use and attentional engagement in everyday life: The role of formulation
Source: PLoS One. 2026 Oct 6;21(10):e0359652. doi: 10.1371/journal.pone.0359652 (PMC13641381; doi:10.1371/journal.pone.0359652)
Supplement: Supplementary file 2 [file pone.0359652.s003.docx]

**Responses to Reviewers**

Reviewer 1:

**Comment 1.** In this study the authors examine the relationships of use and non-use of OC, and of different formulations of OCs, with several attention-related measures among approximately 350 young OC users and over 2200 non-users. The authors found no relationships between use of different formulations of OCs, including different doses, hormonal activities, dosing schedules, and attention measures. The study questions and findings are novel and of clinical and public health interest; the manuscript in general is well-written and the research well-conducted. Below are a few suggestions and comments for the authors.

**Response:** We thank the reviewer for their kind comments on our manuscript. We address their suggestions below.

**Point 1.** In general, the manuscript is quite long, and the introduction in particular is very long and somehow overdetailed; I think it could be shortened, and part of the content could be moved to the discussion, in order to increase readability and effectively illustrate the study rationale and aims. For example, the authors report in very much detail methods and results of two prior studies on the relation (or lack of) between OC use and mind wandering: these two paragraphs (page 4) could be quite shortened, and details omitted/reduced or moved to the discussion. The same with respect to the authors’ previous study described at pages 5-6: could this paragraph be shortened to just report the main results, and perhaps describe the methods in the discussion, if needed?

**Response:** We thank the author for this suggestion. We have removed some detail from the description of prior studies assessing OC use and mind wandering, and OC use and boredom/flow. We have shortened these sections in the Introduction (see p. 3-5).

**Point 2.** In addition: Introduction, page 3, beginning of first paragraph, “Oral contraceptives are a type of hormonal contraceptive composed of ethinyl-estradiol and a synthetic form of progesterone known as progestin”. This claim is somehow imprecise, as this description corresponds to combined oral contraceptives, while progestin-only pills are also oral contraceptives. Please, reformulate.

**Response:** We thank the reviewer for bringing this to our attention. We have amended this sentence to be inclusive of both combination OCs and progestin-only pills: “Oral contraceptives are a type of hormonal contraceptive composed of ethinyl-estradiol **and/or** a synthetic form of progesterone known as progestin.” (p. 3)

**Point 3.** Introduction, page 3, second paragraph: I think the statement “This is (…), of which a key characteristic is rumination, a conscious experience that overlaps with mind wandering” needs a reference.

**Response:** We thank the reviewer for their attention to detail. However, in shortening the Introduction this statement has removed.

**Point 4.** Introduction, pages 3-4, “Regardless, the similarities between the two concepts provide a basis for expecting OC use to be related to increases in mind wandering and perhaps, more generally, reduction in sustained attention”. The rationale to justify the possible link between OC use and reduced attention seems a bit stretched (i.e., OC use is plausibly related to depression > depression is characterized by rumination > rumination is somehow similar/same as mind wandering). Please, consider reformulating this paragraph; I feel that the provided evidence of a link between cognition and OC use, along with lack of studies specifically focused on attention, is enough for justifying the current investigation. The link between depression, rumination, and attention could instead be discussed in result interpretation in the discussion section.

**Response:** We thank the reviewer for this suggestion. We understand this logic might be unwieldy. We have removed the mind wandering and rumination relation as a rationale for studying OC use and attention and replaced it with the notion that few studies have examined OC use and sustained attention (see p. 3):

“As of this writing, just two studies examined the relation between OC use and mind

wandering.”

**Point 5.** Introduction, page 7, “This made us wonder if OC users and non-users may similarly differ once we accounted for the dosing schedule and/or the estrogenic, progestational, and androgenic activity of the OC used.” In addition to the results of the cited studies, the evidence for different OC formulations being related to different depression risks provides an additional rationale for the current study.

**Response:** We agree and have integrated this into the rationale for the study (see p. 5):

“It is possible that different OC formulations may act differently on the brain and produce different behaviours as evidenced by different OC formulations being associated with varying risks of developing depression.”

**Point 6.** Introduction, page 7, second paragraph, “To address this limitation, in the present study we collected data regarding OC brand as well as duration of OC use in order to determine whether there were effects of specific formulations on attentional engagement.” The authors refer to duration of OC use, but, to my understanding, it was not in fact considered in the analyses, although it may be an important moderator in the relationship.

**Response:** When we cleaned the data on the length of OC use, only 35 participants (of the 253 who provided their brand) provided a clear length of time they had used OCs. The remaining participants either did not report the length of time they had used OCs or gave an ambiguous response (e.g., “since 9^th^ grade”) from which we would have had to make assumptions in order to determine the length of time OCs were used. Nevertheless, for exploratory purposes we correlated the length of OC use with our attention measures of interest and found no significant correlations (see p. 8):

“Only 35 participants provided a clear response in terms of the length of time they had used

OCs. As such, we were underpowered to length of use as a mediator or moderator in the

subsequent analyses. Exploratory analyses revealed no significant correlations between length of

use and our attention measures (all ps > .062).”

**Point 7.** Methods, page 8, chapter “Participants”: this chapter is quite long and hard to follow: could the authors consider reporting selection procedure and numbers in a figure instead, and summarize this chapter? For example, given that most of the numbers are already reported in Table 1, the authors could just add a row with the final number of remaining participants per semester to table 1 (Please, also add a reference to Table 1 in the text).

**Response:** We thank the reviewer for this suggestion. We have shortened the participants section by adding additional information to Table 1. We have also included a reference to Table 1 in the text (see p. 7 and p. 9-10).

**Point 8.** Results, page 15: the first two paragraphs belong more to the methods than to the results section.

**Response:** We agree the second paragraph of the results is more of a data analysis plan and has moved into methods (see p. 13-14):

“Our analyses consist of two parts: First, we will compare OC users who reported their brand to

OC users who did not report brand to determine if we can collapse these groups into one OC

group. This will allow us to conduct planned comparisons of OC users to non-users in order to

test the replicability of our prior findings. Second, we will conduct analyses examining the

link between OC composition and our trait-level attention measures to determine whether

particular formulations are related to differences in attention. This will allow us to determine

whether collapsing across OC formulation obscures effects of specific formulations.”

**Point 9.** Results, page 15, “second, we compared OC users who reported their brand to OC users who did not report their brand to ensure that those who reported their brand versus not did not did not differ in terms of their attention”. Although intuitive, the authors need to provide a rationale for these analyses (it is probably a methodological step to allow pooling OC users with and without reported OC brand;, if so, it has to be clarified). The same with regard to the first sentence at page 21. (please, also note the repetition “did not” in the reported sentence).

**Response:** We thank the reviewer for their attention to detail and agree that a rationale should be provided. This rationale is now included (p. 13):

“First, we will compare OC users who reported their brand to OC users who did not report brand

to determine if we can collapse these groups into one OC group.”

**Point 10.** Results, page 15: the meaning/rationale for correlation analyses reported in Table 3 is unclear.

**Response:** We thank the reviewer for bringing this to our attention. We provide these correlation coefficients by OC status as these coefficients will inform the slopes and directions of the later regression analyses. We now state this in the manuscript (p. 14):

“We provide correlation coefficients split by OC status as these will inform the directions of the

later regression analyses.”

**Point 11.** Table 2 is pretty long and hard to read: is there a specific reason why the authors decided to report the measures split by semester rather than pooled together?

**Response:** We split the measures by semester due to the possibility that the measure means may differ by semester. However, since we did not analyze the data by semester this is not critical. As such, we have simplified Table 2 to include the descriptive statistics by OC group only and moved the table including OC group and semester to Supplementary Materials (see p. 14-15):

“Descriptive statistics for each measure as a function of group (OC vs. Non-OC) are presented in

Table 2. Descriptive statistics as a function of group and semester of data collection are included

in Supplementary Materials.”

**Point 12.** Results, page 18, chapter “Describing OC Composition”, “We were able to identify the formulation for all 33 brands reported. Some formulations were branded under multiple names so although 31 different brands were reported,..”. Was the number of reported brands 33 or 31? In table 4 I am able to count 29 brands… Please, solve this inconsistency.

**Response:** We thank the reviewer for bringing this to our attention. We have double checked, and there were 31 different brand reported. This has been corrected in the manuscript and we have ensured there 31 unique brand names listed in Table 4.

**Point 13.** Table 4, regarding estrogenic activity/level: why does estrogenic activity 30 correspond to level “intermediate low” for some brands (first row), but “intermediate high” for others (second row)?

**Response:** We thank the reviewer for their attention to detail. We have double checked all the values provided in the Table 4. This has been corrected (see p. 22).

**Point 14.** Results, page 24, “estrogenic activity was a significant positive predictor, such that more estrogenic activity was associated with more deliberate mind wandering.” This seems incorrect, based on table 4, as the regression coefficient is negative (-0.04), indicating more estrogenic activity associated with less deliberate mind wandering.

**Response:** We thank the reviewer for bringing this to our attention. When we double checked the brands (Point 12), some participants were inadvertently dropped from some analyses. As such, when we re-ran the regressions with those participants included the results slightly changed. One such result was this regression predicting deliberate mind wandering. The estrogenic activity coefficient should be negative, but it is no longer significantly correlated with deliberate mind wandering (p = .115).

**Point 15.** Results, Table 6: do the regression models include any covariate?

**Response:** No the regression models do not include any covariates.

**Point 16.** Results, page 29: as the rationale for comparing OC users who did vs did not report OC brand was to allow collapsing the data (rather than in response to a true study question), I suggest shortening or even moving this section to supplementary material. If instead these results are meaningful in relation to a specific research question, this should be clearly explained.

**Response:** The rationale for comparing OC users who reported brand vs. did not, was to allow collapsing of data – but this collapsing of data was an attempt to replicate our prior work. Replicating our prior work before analyzing the data by OC formulation is important because it allows us to test whether collapsing across formulation obscures effects. As such, it should remain in the main manuscript.

**Point 17.** Results, page 30, last paragraph: in addition to a nominal difference in boredom proneness there seems to be also a nominal difference in internal flow (p=0.045).

**Response:** While the difference between OC users and non-users on both internal and external flow is p < .05 – this result is no longer statistically significant once we apply the Bonferroni correction. We interpret the difference in boredom proneness as nominal and note that as this effect has been observed before (Smith et al., 2026). In prior work, we did not observe significant differences between groups in internal or external flow and as such, the difference in this sample is likely spurious.

**Point 18.** In the discussion section I suggest acknowledging study limitations. For example, the population of the study is quite selected, as it consists of undergraduate psychology students. Also, the authors did not consider possible confounding: for example, could duration of use of OC (or previous use of same/other contraceptives) have an impact on the studied relations?

**Response:** We thank the reviewer for this suggestion. A paragraph on future directions and limitations has been added to the Discussion (p. 32-33):

“This work suggests several future directions. First, this study included a sample of

undergraduates who reported their sex as female. Since undergraduates can be a homogenous

sample in terms of age, education, and socioeconomic background, it is possible that our sample

is not representative of OC users more generally. Future work could utilize platforms that

recruit a more heterogenous sample (e.g., Prolific) and examine how the results compare to

those of the present study. Second, we did not account for several possible confounding factors.

While we would have preferred to control for variables that are known to vary with OC use (e.g.,

depression, duration of OC use), we opted not to as once we split the data by OC formulation

many groups were already underpowered and controlling for additional factors would have

decreased the power further. Future work could recruit larger samples in order to be able to

control for possible confounding factors without compromising power. We provide suggestions

for recruiting these large samples in Smith, Smilek, and Fenske (in prep). Third, as we have noted

earlier, the goal of the present study was to determine whether collapsing across OC formulation

obscured differences in everyday attention that would emerge once formulation was included.

The goal was not to determine whether differences in OC formulation were related to

differences in everyday attention. The latter question would require a much larger sample in

order adequately power groups once the sample was split by the different aspects of OC

formulation (e.g., dosing schedule, progestin generation). Nevertheless, this is a question worthy

of investigation and could be examined in future work.”

**Point 19.** There are some typos here and there (e.g., page 7: “They found that while women using OCs did not significantly differ from naturally cycling women, but women using monophasic OCs did significantly…”)

**Response:** We thank the author for pointing this out. We have re-phrased this sentence for clarity (p. 5-6):

“When they collapsed across OC formulation, they found that women using OCs did not

significantly differ from naturally cycling women, but when OC formulation was considered,

women using monophasic OCs significantly differed from naturally cycling women such that

monophasic OC users outperformed naturally women.”

**Point 20.** Page 19, “see footnote 5” should probably be “see footnote f”.

**Response:** We thank the author for their attention to detail. This has been corrected.

**Point 21.** In Table 4, is estrogenic level “intermediate low” same as “low intermediate”?

**Response:** Yes, we have corrected the labels in Table 4 to be consistent.

Reviewer 2:

**Point 1.** Clearly differentiate between the aim of the study and the objectives

**Response:** We thank the reviewer for this suggestion. We have separated the aim from the objective in the present study section (p. 6-7):

“…in the present study we aimed to examine whether collapsing across OC formulation obscured

differences in everyday attentional engagement.”

“Our main objectives were as follows: 1) replicate the overall null relations between OC use and everyday attentional engagement we reported previously; and 2) then examine the role of OC formulation to determine whether collapsing across OC formulations obscured effects of specific formulations.”

**Point 2.** How is the sample taken from different sources, maintaining the homogeneity?

**Response:** The sample was not taken from different sources. All data was collected at the University of Waterloo via the Pre-screen and Mass Testing surveys (p. 7):

“Participants in the secondary dataset were undergraduate students enrolled in at least one psychology course at the University of Waterloo who indicated their sex as female. In exchange for completing the Pre-screen and Mass Testing surveys (see below), participants received partial course credit.”

**Point 3.** Kindly provide a clear justification for the sample size calculation, including the formula/software used, assumptions, and statistical parameters (e.g., confidence level, power, effect size, and margin of error..

**Response:** This study utilized secondary data and as such, we did not have control over the sample size. This was stated on p. 7:

“Participants in the secondary dataset were undergraduate students…”

**Point 4.** Clearly describe the sampling technique and justify why this method was appropriate for the study population.

**Response:** This study utilized secondary data and recruited undergraduate students enrolled in at least one psychology course at the University of Waterloo. Since the aim of the present study was to determine whether collapsing across OC formulation in our prior work (Smith et al., 2023) obscured differences in everyday attentional engagement, we aimed to collect data using the same recruitment techniques as that prior work. This is now stated on p. 7:

“Since our goal was to determine whether our prior work collapsing across OC formulation obscured differences in everyday attentional engagement, we aimed to collect data using the same recruitment techniques.”

**Point 5.** State the inclusion and exclusion criteria used for participant selection.

**Response:** Inclusion and exclusion criteria can be found in the data cleaning section (p. 8-9) and in Table 1.

**Point 6.** Please provide the complete dataset to enable verification and improve the transparency and reproducibility of the findings.

**Response:** We provided a link to the anonymized data and data analysis scripts on p. 14:

“Anonymized data and analysis scripts are available at: https://osf.io/myad4/overview?view_only=e16209741cb84e4db794e3ba3e00c103”

**Point 7.** Kindly specify the statistical software used, including the software name and version.

**Response:** We have provided details on the software, version, and R packages used to clean and analyze the data on p. 14:

“All analyses were conducted using R49. To perform Null Hypothesis Significance Tests we used R (version 4.4.1) and utilized the psych, stats, Hmisc, ez, and basic R packages.”

**Point 8.** Please share the complete statistical analysis output (software output files. to facilitate verification of the reported results and statistical procedures.

**Response:** Analysis code and outputs are available on the Open Science Framework (OSF). The link was provided on p. 14.

**Point 9.** Describe the data cleaning and preprocessing procedures, including the handling of missing values, outliers, and data validation before analysis.

**Response:** Data cleaning procedures (including the handling of missing values and data validation before analysis) were provided in the method section under data cleaning (p. 8-9). We did not exclude any data on the basis of outliers.

**Point 10.** State the statistical assumptions assessed before conducting the analyses and report the corresponding diagnostic test results.

**Response:** We thank the reviewer for this suggestion. We understand the statistical assumptions of the analyses included in the paper and have ensured we ran the proper test based on the characteristics of the data. We do not include them in the main manuscript as this would make the results section unnecessarily lengthy.

For your interest, we provide the statistical assumptions below:

- Spearman correlations conducted as the data was not normally distributed but was ordinal or continuous.
- Regression analyses assume (1) a linear relationship between the dependent and independent variables, (2) independent residuals, (3) normally distributed data, (4) equal variance, and (5) no multicollinearity. We did violate the assumption of normality but Li et al. (2012) provided evidence that linear regression can provide good estimates even if the normality assumption is violated.
- ANOVA assume (1) independent observations, (2) normally distributed data, (3) homogeneity of variance, and (4) continuous dependent variables. We again violated the assumption of normality, however, again several studies with simulated data have suggested that ANOVA is robust to this type of violation (e.g., Blanca et al., 2017; Schmider et al., 2010).
- Planned comparisons were conducted using t-tests and a Welch (or Satterthwaite) approximation to account for the unequal variances/sample sizes.

**Point 11.** Justify why you have used these statistical methods

**Response:** Again, we do not provide justification for the statistical methods in the main manuscript but we provide justification for each method below.

- Spearman correlations were used when we wanted to understand how strongly and in what direction two variables moved together.
- Regression analyses were used when we wanted to know the mathematical relationship between one or more independent variables (predictors) and a dependent variable (outcome). Predictors tell us their unique relation with the dependent variable, holding the other predictors constant.
- ANOVA was used when we wanted to know if there was a significant difference between the means of three or more groups.
- Planned comparisons were used when we wanted to know if the difference between the averages of two groups was statistically significant.

**Point 12.** Show both types of datasets in a diagram

**Response:** We are unclear what the reviewer is requesting here. We provide several figures showing the data split by the groups (e.g., Figure 3 shows our dependent variables as a function of dosing schedule, Figure 4 shows our dependent variables as a function of progestin generation, Figure 1 shows our dependent variables as a function of whether or not OC users reported their brand, and Figure 2 collapses across OC formulation to compare OC users and non-users).
